# Applications of Ferro-Nanofluid on a Micro-Transformer

**DOI:** 10.3390/s100908161

**Published:** 2010-08-31

**Authors:** Tsung-Han Tsai, Long-Sheng Kuo, Ping-Hei Chen, Da-sheng Lee, Chin-Ting Yang

**Affiliations:** 1 Department of Mechanical Engineering, National Taiwan University, No. 1, Sec. 4, Roosevelt Rd., Taipei 10617, Taiwan; E-Mails: f91522531@ntu.edu.tw (T.-H.T.); d94522017@ntu.edu.tw (L.-S.K.); 2 Department of Energy and Refrigerating Air-conditioning Engineering, National Taipei University of Technology, No.1, Sec. 3, Chung-hsiao E. Rd., Taipei 10608, Taiwan; E-Mail: f11167@ntut.edu.tw (D.-S.L.); 3 Department of Mechanical and Computer-Aided Engineering, St. John’s University, No. 499, Sec. 4, Tam-king Rd., Tamsui, Taipei 25135, Taiwan; E-Mail: ctyang@mail.sju.edu.tw (C.-T.Y.)

**Keywords:** MEMS transformer, ferrofluid

## Abstract

An on-chip transformer with a ferrofluid magnetic core has been developed and tested. The transformer consists of solenoid-type coil and a magnetic core of ferrofluid, with the former fabricated by MEMS technology and the latter by a chemical co-precipitation method. The performance of the MEMS transformer with a ferrofluid magnetic core was measured and simulated with frequencies ranging from 100 kHz to 100 MHz. Experimental results reveal that the presence of the ferrofluid increases the inductance of coils and the coupling coefficient of transformer; however, it also increases the resistance owing to the lag between the external magnetic field and the magnetization of the material.

## 1. Introduction

In recent years, on-chip transformers have been widely used and in highly demand for small-size DC/DC converter applications. These converters are mainly applied in portable electronic products such as mobile phones, notebooks, and e-paper [1–27]. Many on-chip transformers with different magnetic cores have been investigated. Details of the unfavorable factors such as parasitic capacitance, ohmic loss, and substrate loss of these transformers have been discussed in some detail [10–12,16,18,26,27]. Other than the above factors, a portion of the papers have indicated that the core loss from the “solid” magnetic core significantly affects the performance of the transformers. Solutions for the core loss were further discussed in [1,14,19]. On the contrary, only a handful of papers have discussed transformers with “liquid” magnetic cores. These liquid-core transformers with their distinguishing features of low electric conductivity and the super-paramagnetism of oil-based ferrofluids, have became a solution to the core losses of eddy currents and hysteresis losses. In this study, a ferrofluid is applied as a “liquid” magnetic core in the transformer, and the performance of this transformer is compared to that of a transformer with an air core.

## 2. Experimental Section

### 2.1. Fabrication of Oil-based Fe_3_O_4_ Nanofluids

The oil-based Fe_3_O_4_ nanofluids used in this study were composed of Fe_3_O_4_ nanoparticles, surfactant and a mixture of diesel oil and polydimethylsiloxane (PDMS). The Fe_3_O_4_ nanoparticles were fabricated by the co-precipitation method for an efficient reaction time and production. The corresponding chemical reaction can be expressed as:

(1) $${\mathrm{Fe}}^{2+}+2{\mathrm{Fe}}^{3+}+8OH^{-}\to {\mathrm{Fe}}_{3}O_{4}+4H_{2}O$$

**Figure 1.**
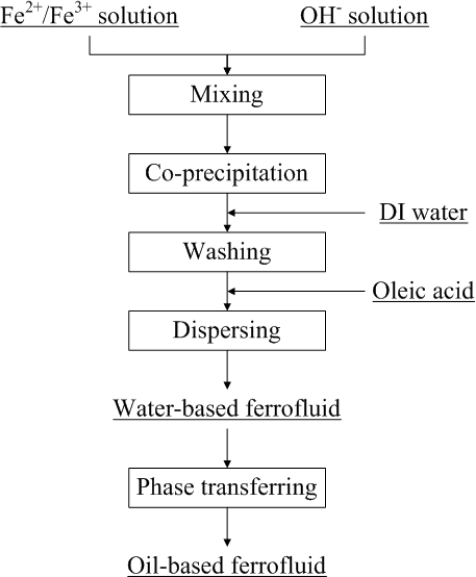
The flow chart of the precipitation procedure.

The flow chart of the precipitation procedure is shown in Figure 1. In the first step, FeCl_2_·4H_2_O and FeCl_3_·6H_2_O with a mole ratio of 1:2 were dissolved in 100 mL D.I. water. In another beaker, a suitable amount of NaOH was dissolved in 400 mL of D.I. water. Then the Fe^2+^/Fe^3+^ solution from step one was slowly poured into the NaOH solution under stirring at 500 rpm. The Fe_3_O_4_ nanoparticles were formed immediately along with a black color change. After the precipitation reaction, the pH of the solution should be maintained between 10 and 12. There are some unwanted impurities and ions present in the solution that should be washed out. After keeping the solution static for a short period of time, the Fe_3_O_4_ nanoparticles were precipitated, leaving the top of the solution transparent. A permanent magnet was used to accelerate the separation of Fe_3_O_4_ nanoparticles and the solution. The washing process was repeated several times to remove unwanted impurities and ions. About 150 mL of water was left in the beaker after the washings. In order to prevent the Fe_3_O_4_ nanoparticles from aggregation and at the same time modify the surface of the Fe_3_O_4_ nanoparticles, oleic acid was added as a surfactant. Oleic acid is insoluble in water, and that is why ammonia was used to modify the functional group of oleic acid and that dissolves in water. The chemical reaction of the modification process is expressed as:

(2) $$∼\mathrm{COOH}+{\mathrm{NH}}_{4}\mathrm{OH}\to ∼{\mathrm{COONH}}_{4}+H_{2}O$$

**Figure 2.**
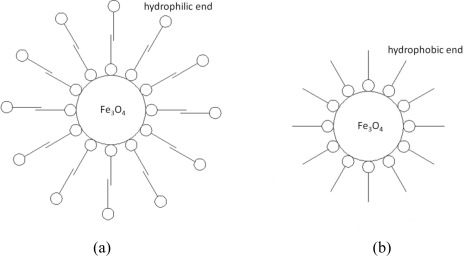
The adsorption model showing the relation between the surfactant and the particle: (a) Model for water-based Fe_3_O_4_ nanofluid (b) Model for oil-based Fe_3_O_4_ nanofluid.

There are two ends in an oleic acid molecule, each with a different property. The *∼COONH_4_* end is hydrophilic, and the hydrocarbon end is hydrophobic. After adding ammonia and oleic acid sequentially, the solution was heated to 80 °C under constant stirring for 2 hours. After this step, a water-based Fe_3_O_4_ nanofluid was obtained. The hydrophilic end of oleic acid was attached to the surface of Fe_3_O_4_ nanoparticles, which forms the first layer of surfactant as shown in Figure 2(a). The hydrophobic end of the fixed oleic acid particles were further connected to other oleic acid molecules in the solution to form a second layer of surfactant. The two-layer structure provides stability to Fe_3_O_4_ nanoparticles, and uniforms the distribution of the nanoparticles in water.

**Figure 3.**
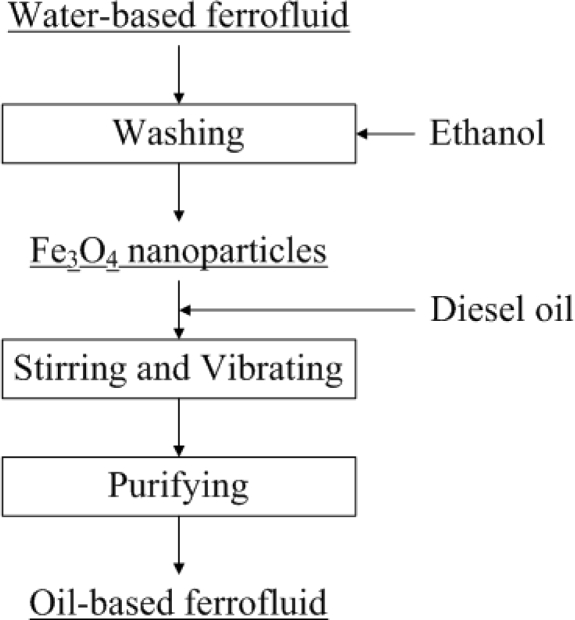
The procedure of phase transferring from water to oil.

The phase of the fluid can be changed by applying the phase-transfer method. The phase-transfer process is shown in Figure 3. In this study, ethanol served as a medium that can dissolve in both water and diesel oil. A sufficient amount of ethanol was added to the water-based Fe_3_O_4_ nanofluid, the solution was stirred for 3 minutes. After keeping the solution static for a period of time, the Fe_3_O_4_ nanoparticles were precipitated. A permanent magnet was applied to the nanofluid to accelerate the separation process. After repeatedly washing out the outer layer surfactant of the nanoparticles with ethanol, single-layered hydrophobic Fe_3_O_4_ nanoparticles were obtained, as shown in Figure 2(b). In the final step, required amount of diesel oil was mixed with the Fe_3_O_4_ nanoparticles, and treated in an ultrasonic sieving for 2 hours. The residual water and ethanol were removed by magnetic separation and baking methods and the oil-based Fe_3_O_4_ nanofluid was thus obtained.

**Figure 4.**
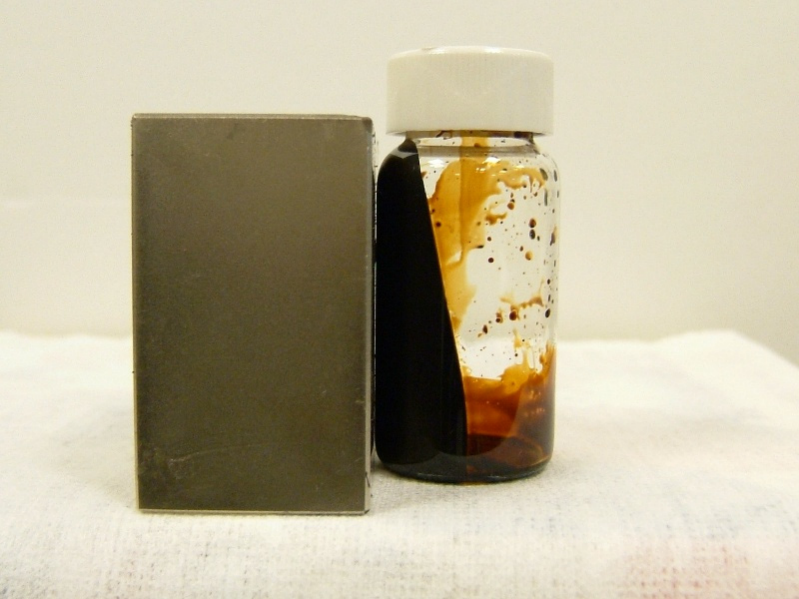
The magnetic effect on the ferrofluid.

**Figure 5.**
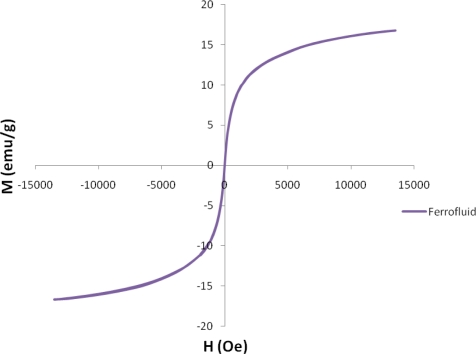
The magnetized curve of 1M ferrofluid measured by a VSM.

Figure 4 shows the effect of a magnet on the ferrofluid. It is observed that the magnetic effect on the ferrofluid is sufficient to overcome the gravitational force. Due to the surfactant, the Fe_3_O_4_ nanoparticles were well dispersed in the solution even under a strong magnetic field. The resulting magnetized curve of the 1 M ferrofluid measured by a vibrating sample magnetometer (VSM) is shown in Figure 5. The ferrofluid illustrates the characteristic of super-paramagnetism. The saturated magnetization of 1M ferrofluid is 16.7 emu/g.

### 2.2. Fabrication of MEMS Transformer

**Figure 6.**
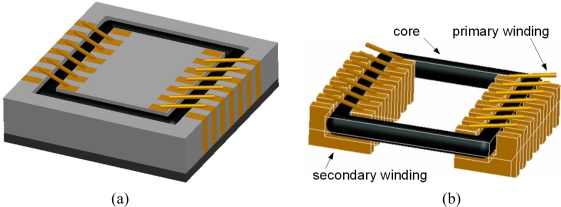
The schematic diagram of solenoid type transformer: (a) the whole structure; (b) the perspective diagram.

**Figure 7.**
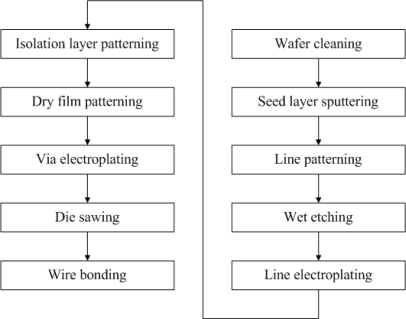
The fabrication process of the MEMS transformer.

In this study, a solenoid type transformer was fabricated on a wafer by the MEMS process. The schematic diagram of solenoid type transformer is shown in Figure 6. The fabrication process of the MEMS transformer is shown in Figure 7.

Firstly, a wafer was cleaned and coarsened by a reactive ion etch (RIE) to remove impurities and at the same time increase the adhesion between the wafer and other materials. The Cr/Cu seed layer is sputtered on the wafer by a physical vapor deposition (PVD) process. The lines are patterned by the EPG-512 photoresist. The undercover metal layer was etched by the corresponding etchants. Then the photoresist was removed. To reduce the resistance of lines on the wafer, a 6-μm Cu layer and a 1-μm Au layer were electroplated on the seed layer sequentially. A 12-μm-thick light-sensitive polyimide was coated onto the wafer and the via holes were patterned for insulation between the channel and bottom structure. Two layers of dry film were attached onto the wafer by a dry film machine. Via holes and channels were patterned, and 200-μm-high metal vias were electroplated onto the wafer. The wafer was then cut into small pieces according to the cutting lines.

**Figure 8.**
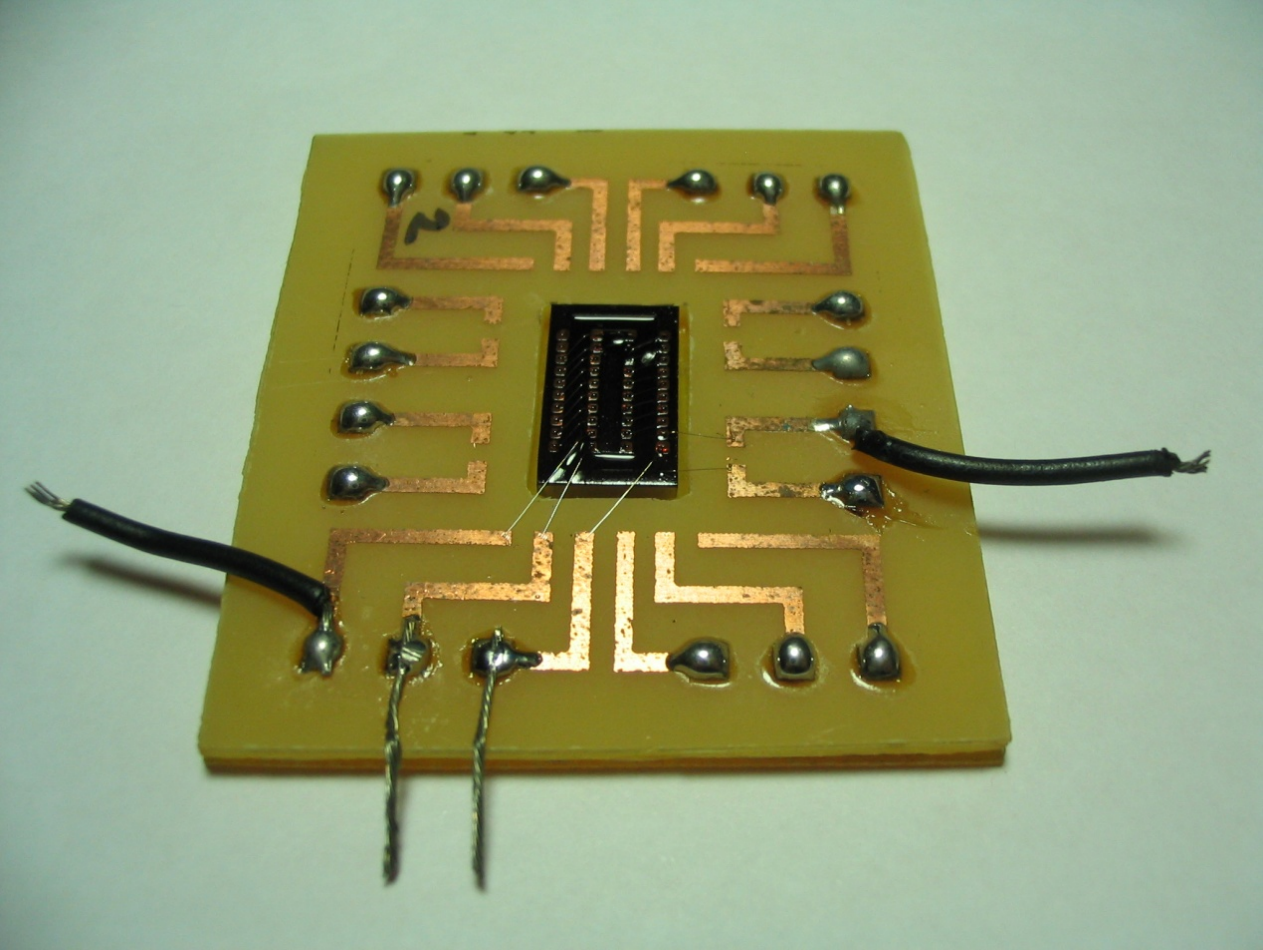
The measuring sample of MEMS transformer.

Finally, the Al wires were bonded to the vias to construct the top structure of transformer, and the wafer was bonded on a printed circuit board (PCB). The final testing sample is shown in Figure 8.

The controlled parameter of this study was the magnetic core of the transformer. A traditional solid magnetic core transformer has hysteresis properties that causes energy losses, especially at high frequencies. For this reason, the to reduce the hysteresis loss solid magnetic core is substituted with an oil-based Fe_3_O_4_ nanofluid which possesses the property of super-paramagnetism. Moreover, due to its super-paramagnetism properties and the higher permeability than air, a Fe_3_O_4_ nanofluid magnetic core transformer is expected to have a better performance than an air core transformer. In this study, air and Fe_3_O_4_ nanofluid were used as magnetic cores of two transformers. Self-inductance (*L*), leakage inductance (*L_sc_*), coupling coefficient (*K*), resistance (*R*) and quality factor (*Q*) were measured by a precision impedance analyzer (4294A, Agilent Technologies) with a frequency range of 100 kHz to 100 MHz.

## 3. Results and Discussion

**Figure 9.**
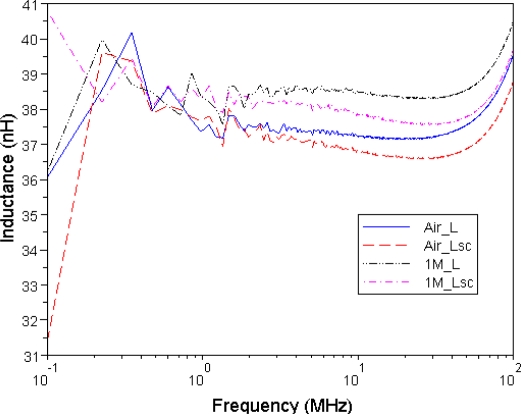
The self-inductances (L) and leakage inductances (L_sc_) of coils of MEMS transformer with the air core and magnetic core of 1 M Fe_3_O_4_ nanofluid.

Figure 9 shows the self-inductances and leakage inductances of two MEMS-core transformers, each with an air core and a 1 M Fe_3_O_4_-nanofluid magnetic core. The graph shows that the self-inductance and leakage inductance increased with the presence of 1 M Fe_3_O_4_ ferrofluid. Between frequencies of 100 kHz and 20 MHz, the inductances decrease slightly due to the skin effect. At frequencies over 20 MHz, the self-inductance gradually increases as it approaches the maximum inductance at the resonance frequency.

**Figure 10.**
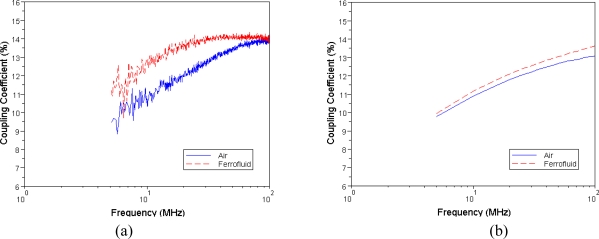
The coupling coefficient of MEMS transformers with the air core and magnetic core of 1 M Fe_3_O_4_ nanofluid: (a) measured data; (b) simulated data.

**Figure 11.**
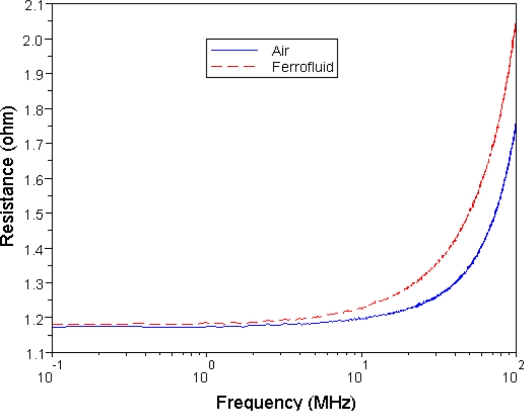
The resistance of coils with the air core and magnetic core of 1 M Fe_3_O_4_ nanofluid.

Figure 10 shows the measured and simulated results of coupling coefficient of the two MEMS transformers. The coupling coefficient showed a slight increment by substituting the air core with a nanofluid core. The above results showed that nanofluid magnetic cores are able to improve the inductance and coupling coefficient of transformers. As Figure 11 shows, however, the resistance of each pattern increases as a function of frequency. In addition to that, the resistance increases as the concentration of Fe_3_O_4_ increases. This is because when the magnetic core is magnetized at a high frequency; there will always be a lag on the magnetization process of the material behind an external magnetic field.

**Figure 12.**
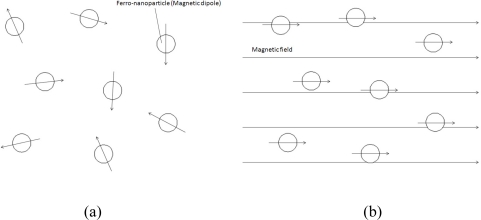
The macroscopic view of ferro-nanoparticles: (a) without a magnetic field; (b) with a magnetic field.

**Figure 13.**
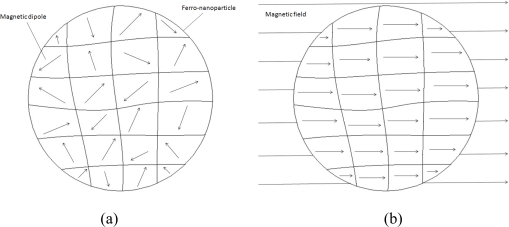
The microscopic view of a ferro-nanoparticle: (a) without a magnetic field; (b) with a magnetic field.

The reasons for the lag are speculated to be as follows:

1. From the macroscopic point of view, a nanoparticle can be considered as a small magnetic dipole, which is shown in Figure 12. The direction of the magnetic dipoles was originally random. When an external magnetic field is applied, the direction of magnetic dipoles will turn to the direction of the external magnetic field and form a non-zero total magnetic dipole moment. Once the external magnetic field is removed, the direction of magnetic dipoles would return to random distribution with a zero total magnetic dipole moment due to Brownian motion in a short period of time. This recovering period of time is called “Brownian relaxation time” [28].
2. From the microscopic point of view, a nanoparticle is composed of numerous magnetic dipole moments, as shown in Figure 13. These magnetic dipole moments were also influenced while an external magnetic field was applied or removed. The recovering time of these magnetic dipole moments is called “Neel relaxation time” [28].

Therefore, when frequency of alternate external magnetic is high enough, in other words, the alternate time of magnetic field is shorter than the relaxation time, a lag would occur and result in the increase of resistance.

At lower frequencies, external magnetic field and the magnetization of the material can be regard as if they were proportional to each other through scalar permeability. In contrast, at higher frequencies, the external magnetic field and the magnetization of material will react with each other to some extent, so the permeability can be expressed as a complex number given by the function below:

(3) $$\mu^{*}=\mu \prime -j\mu \prime \prime$$

The real part of the complex permeability *μ′*, which has the same phase as the magnetic field, represents the stored energy coefficient when the material is magnetized. The imaginary part of the complex permeability *μ″*, which has a phase of 90 degrees delay to the magnetic field, represents the consumed energy coefficient of magnetized material. The inductance of a solenoid-type inductor is expressed as follows:

(4) $$L=\frac{\mu^{*}N^{2}A}{l}$$

where *N* is the turns of the coil, *A* is the cross-sectional area of the solenoid, and *l* is the length of the solenoid. Therefore, the impedance of a real inductor with resistance *R* is expressed by:

(5) $$Z\equiv R+j\omega L=R+\omega \frac{\mu \prime \prime N^{2}A}{l}+j\omega \frac{\mu \prime N^{2}A}{l}$$

The imaginary portion of the complex permeability will reflect a dramatic change in resistance as the frequency increases. The quality factor *Q,* which is defined as the ratio of the inductive reactance to the resistance, becomes:

(6) $$Q\equiv \frac{\text{Im}(Z)}{\text{Re}(Z)}=\frac{\omega \mu \prime N^{2}A}{Rl+\omega \mu \prime \prime N^{2}A}$$

**Figure 14.**
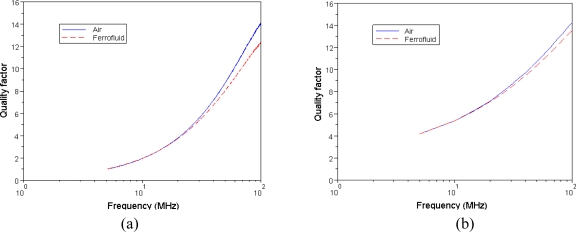
The quality factor of coils with the air core and magnetic core of 1 M Fe_3_O_4_ nanofluid: (a) measured data; (b) simulated data.

Figure 14 shows the measured and simulated results of quality factor corresponding to coils with different magnetic cores. The increment of resistance is steeper than the increment of inductance with Fe_3_O_4_ nanofluid as core. This result leads to the fact that the quality factor decreases with presence of the ferrofluid.

## 4. Conclusions

In this study, the performance of the MEMS transformer of a ferrofluid-magnetic core was investigated. The experimental results revealed that with Fe_3_O_4_ nanofluid as core, the inductance of coils and coupling coefficient of transformer were improved. Nonetheless, due to lag between the magnetization of material and the external magnetic field, the resistance increased to a greater extent, even faster than inductance, hence resulted in a low quality factor. On the other hand, the ferrofluid could be applied as a carrier for delivering ferro-nanoparticles into microchannels. Furthermore, by repeatedly adding ferrofluid and removing the base fluid, a solid magnetic core can be obtained. This fabrication process of solid magnetic core has a lower thermal budget than the sputtering and electroplating processes, and it is compatible with the MEMS process.
